# Transition state theory demonstrated at the micron scale with out-of-equilibrium transport in a confined environment

**DOI:** 10.1038/ncomms10227

**Published:** 2016-01-06

**Authors:** Christian L. Vestergaard, Morten Bo Mikkelsen, Walter Reisner, Anders Kristensen, Henrik Flyvbjerg

**Affiliations:** 1 Department of Micro- and Nanotechnology, Technical University of Denmark, DK-2800 Kgs. Lyngby, Denmark

## Abstract

Transition state theory (TST) provides a simple interpretation of many thermally activated processes. It applies successfully on timescales and length scales that differ several orders of magnitude: to chemical reactions, breaking of chemical bonds, unfolding of proteins and RNA structures and polymers crossing entropic barriers. Here we apply TST to out-of-equilibrium transport through confined environments: the thermally activated translocation of single DNA molecules over an entropic barrier helped by an external force field. Reaction pathways are effectively one dimensional and so long that they are observable in a microscope. Reaction rates are so slow that transitions are recorded on video. We find sharp transition states that are independent of the applied force, similar to chemical bond rupture, as well as transition states that change location on the reaction pathway with the strength of the applied force. The states of equilibrium and transition are separated by micrometres as compared with angstroms/nanometres for chemical bonds.

## Abstract (web summary)

Transition state theory has proven to be a powerful tool for the analysis of a number of processes, perhaps most commonly chemical reactions. Here, the authors use transition state theory to model a directly observable, micron scale process—the transport of DNA molecules in a confined environment.

## Graphical abstract

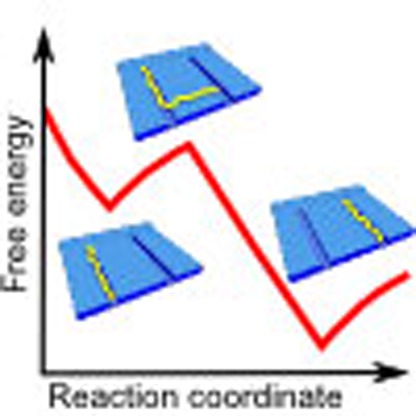

Transition state theory (TST), with its scenario of a reaction pathway through a free-energy landscape (Fig. 1), provides concepts for understanding how thermally activated processes take place. Its development can be traced back to the second half of the nineteenth century^1^, notably to 1889 when Arrhenius proposed his famous empirical relation between the reaction rate *r* of an irreversible chemical reaction and temperature *T*:




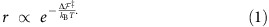




Here 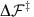 is the height of the free-energy barrier separating the initial state (reactants) and the end state (product), and *k*_B_ is the Boltzmann constant. Theoretical efforts to describe such reactions led to the development of TST in the second half of the 1930s, notably by Eyring, Polanyi, Evans and Wigner^1^,^2^,^3^. TST for elementary chemical reactions assumes, as Wigner summarized it, statistical mechanics, classical motion of atomic nuclei, adiabatically changing electronic states and what has become known as TST's fundamental assumption, fundamental dynamical assumption or no-recrossing assumption. When the reaction process is described by a single reaction coordinate *x*, as in Fig. 1, the no-recrossing assumption states that if *x* crosses the point of maximal free energy—the ‘transition state' *x*^‡^—from left to right, it does not recross from right to left. This is plausible if motion in *x* is inertial, as in chemical reactions between colliding gas molecules.

To investigate the validity of TST, Kramers introduced in 1940 a model, which has become known as ‘Kramers' problem'^4^,^5^. This model relaxes TST's no-recrossing assumption. It considers the reaction to be described by a fictive particle undergoing Brownian motion with more or less friction in the free-energy landscape along the reaction coordinate (Fig. 1). The case of large friction does not model colliding gas molecules, but reactants diffusing in a liquid. It differs from Wigner's TST by having *x* diffuse across *x*^‡^ with multiple recrossing expected from its trajectory of Brownian motion. In many cases Kramers' formalism allows calculation of the proportionality factor between the reaction rate in equation (1) and the Boltzmann factor, but it does not change the exponential dependence on the barrier height in equation (1).

Despite their simplicity, TST and Kramers' model are surprisingly successful at predicting chemical reaction rates, and they are unrivalled at providing conceptual insight into how such reactions occur. Though devised to describe chemical reactions (Fig. 1a), where reaction pathways are measured in fractions of angstroms and reaction times in femtoseconds^6^, their formalism has been extended to processes taking place at timescales and length scales that are orders of magnitude longer. At the nanometre scale the formalism has been applied to rupture of chemical bonds^7^,^8^,^9^,^10^,^11^ (Fig. 1b), protein (un)folding^12^,^13^,^14^ and RNA unzipping^15^ under both constant and time-dependent loads^16^,^17^,^18^; at the micron scale it has been applied to polymers crossing entropic barriers^19^,^20^,^21^,^22^. Han and Craighead notably showed that TST describes the mean waiting time before translocation of a randomly coiled DNA molecule from one micro-groove to another through a nanoslit, driven by an external electric field. The transition state occurs where the external force that squeezes the coil into the slit balances entropic recoil forces^19^,^20^.

We have here replaced the microgrooves of Han and Craighead, in which the DNA assumes a bulk coiled conformation, with nanogrooves that force the molecule to extend linearly and transversely to the axis of propagation (Figs 1c,d and 2). This ensures that the molecule can escape via clearly defined excursions of its end points into the nanoslit (Fig. 2c), leading to a well-defined single reaction coordinate, the DNA strand's extension into the nanoslit (Fig. 2d). The corresponding free-energy landscape for the DNA is (a) one dimensional; (b) tuneable; (c) so large that we can see the DNA moving through it, from one quasi-equilibrium state, over a free-energy barrier, into another quasi-equilibrium state, and so on repeatedly (Fig. 2e); (d) periodic, so each escape is an independent repetition of the same process, which allows us to accumulate good statistics; and (e) so simple that we find a closed formula for transition rates.

We study the translocation of DNA strands between nanogrooves driven by an external flow (Figs 1c,d and 2). For sufficiently weak flow, Brownian motion dominates the dynamics of the DNA to such a degree that its translocation between neighbouring nanogrooves is diffusive^23^. As one increases the flow, and hence its force on the DNA, the system transitions smoothly from the ‘diffusion-dominated regime' to a ‘force-dominated regime' in which translocation is irreversible and described by TST; see below. In the force-dominated regime, Brownian motion still plays a pivotal role by providing the fluctuations that let the DNA cross the entropic barrier separating neighbouring grooves. However, after the DNA has crossed the transition state, translocation is effectively deterministic and dictated by the external force field of the imposed flow.

We show theoretically, using TST, and verify experimentally by measuring the waiting time in each nanogroove that in the force-dominated regime two distinct sub-regimes exist for the transition of the DNA molecule from one groove to the next: (i) for large separation between grooves and high flow speeds, the transition state lies inside the nanoslit (Fig. 1c). Its location is determined by the balance between entropic and drag forces^19^,^20^,^22^ and thus changes with the applied flow speed. (ii) As we decrease the external field (the flow), the location of the transition state moves in the field's direction (downstream), until it reaches the end of the nanoslit. Below the critical field strength at which this happens, the transition state does not move further downstream. It remains fixed at the width of the nanoslit (Fig. 1d). In this previously unobserved low-force (yet force dominated) regime, the transition state is independent of the external field. Both the initial and transition states are here ‘sharp'—that is, the derivative of the free energy with respect to the reaction coordinate is not continuous at these points; it changes value abruptly. This is why the initial and transition states do not move along the reaction coordinate when we alter the external field. The dynamics in the low-force regime is consequently described by the Bell–Evans^7^,^8^,^9^ model for chemical bond breaking under external load.

## Results

### Transition state theory for DNA translocation via a nanoslit

**Figure 2:**
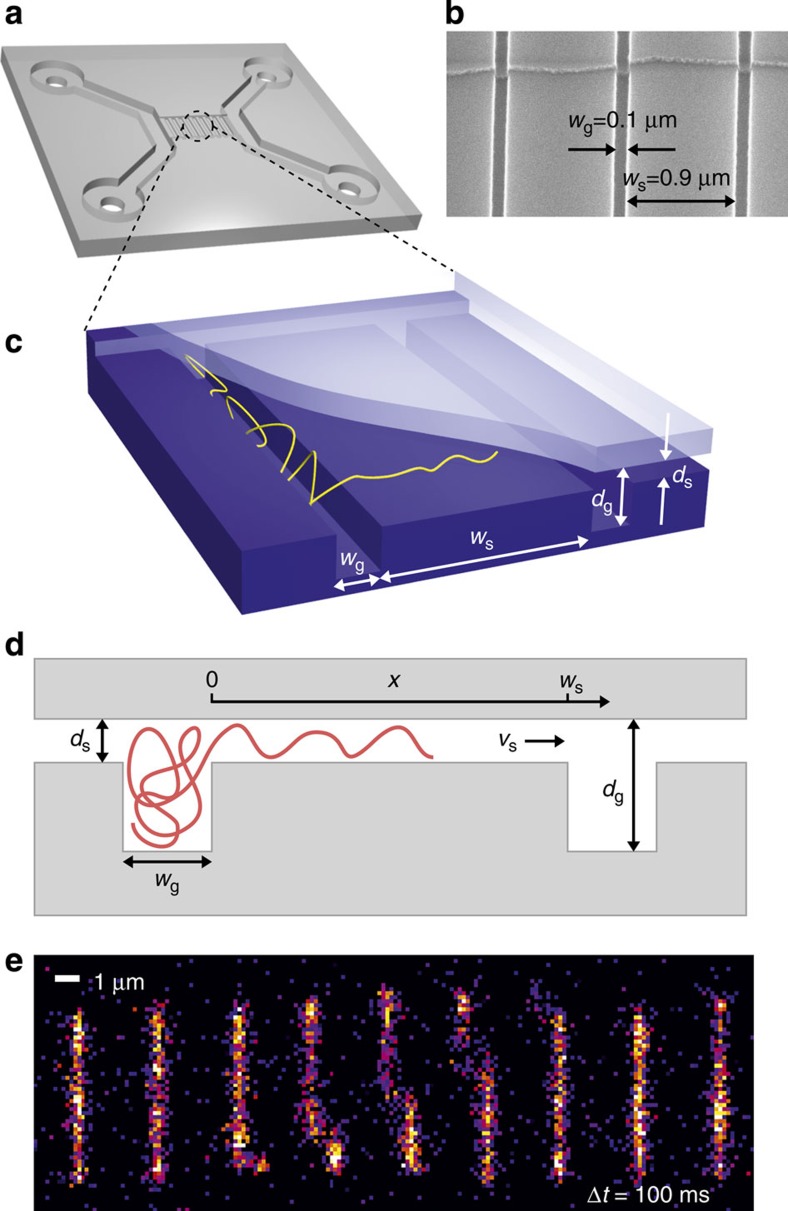
Experimental set-up. (**a**) Schematic drawing of the microfluidic device containing the nanogroove array. A hydrodynamic flow (from left to right) is induced in the chip by imposing a pressure difference Δ*P* over the chip. Fluorescently labelled *λ*-DNA molecules (48.5 kb, *L*_DNA_=21 μm) were introduced into the nanogroove array by the flow. (**b**) Electron micrograph of a section of a nanogroove array. (**c**) Schematic representation of a DNA strand trapped in a nanogroove and attempting to cross the nanoslit separating two grooves. The nanogroove geometry extends the DNA molecule transversally to the flow direction. Consequently, escape into the nanoslit is initiated by an end of the DNA. This vastly simplifies the dynamics compared with other entropic trapping geometries where polymers tend to form herniations inside the nanoslit. (**d**) Same as **c**, but showing a cross-section perpendicular to the nanogrooves. The extent of the DNA molecule's end inside the slit in the direction of the flow is called *x*. The hydrodynamic drag force on the DNA is proportional to *x* and *υ*_s_, where *υ*_s_ is the mean speed of the buffer flow inside the nanoslit. The relevant dimensions of the nanogroove array are the height of the nanoslit, *d*_s_≈50 nm; the total height of a nanogroove plus the nanoslit, *d*_g_≈150 nm; the width of a nanogroove, *w*_g_≈100 nm; and the width of a nanoslit separating two grooves, *w*_s_=0.4, 0.9, 1.9, 3.9 μm. (**e**) Montage of fluorescence images of a DNA molecule performing a sidewinder transition from one groove to the next^21^. The timelapse between consecutive images is 0.1 s. The fluorescence intensity is indicated with false colours. Uneven fluorescence of DNA in nanochannels is due to thermal fluctuations in the density of DNA in channels, where it coils a little as indicated in **c**. The lower fluorescence of DNA where it connects two channels in frames 3–6 (counting left to right) is due to the DNA being stretched in the slit, as indicated in **c**. Frame 7 shows the very last part of the transition between channels/barrier crossing. **a**,**b** and **e** are adapted from ref. 21.

**Figure 3:**
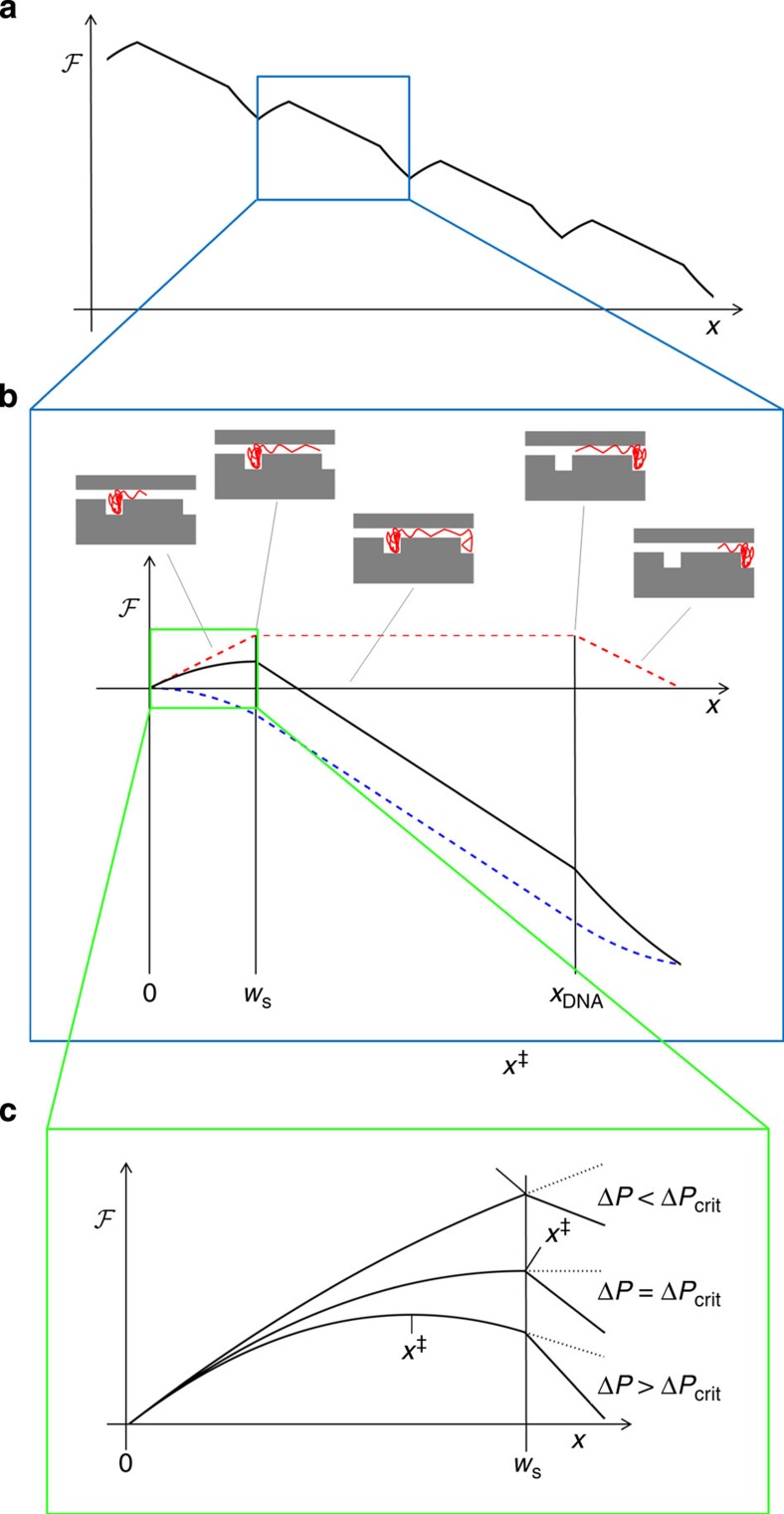
Energy landscape experienced by a DNA molecule in the nanogroove array. (**a**) Free energy 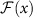 experienced by the molecule when driven by the force field from the buffer flow through a series of entropic traps. (**b**) Free energy 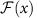 experienced by a the molecule during a single transition between adjacent grooves. Insets show schematic drawings of the physical situation corresponding to five values of *x*. Here *x* is the *x* coordinate inside the nanoslit of the leading end of the DNA strand until that end descends into the next nanogroove. From that point and until the next state of quasi-equilibrium has been reached, further increase in *x* describes the length of DNA that has entered the next nanogroove. Thus, *x*=0 corresponds to the equilibrium state in which the whole strand resides in the left nanogroove. At *x*=*w*_s_ the leading end enters the next nanogroove. At *x*=*x*_DNA_, the trailing end of the DNA leaves the upstream nanogroove. At *x*=*x*_DNA_+*w*_s_, the DNA is again in quasi-equilibrium in the next groove, and the energy landscape repeats itself downstream from there as shown in **a**. Red dashed line: contribution to the free energy due to loss of entropy; blue dashed line: loss of potential energy of the DNA due to higher hydrodynamic drag on the part of the strand inside the nanoslit; black full line: total free-energy difference 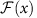. (**c**) Zoom on the energy landscape for 0<*x*≤*w*_s_. The transition state at *x*^‡^≤*w*_s_ is the point with maximal free energy. For Δ*P*≤Δ*P*_crit_, *x*^‡^=*w*_s_; for Δ*P*>Δ*P*_crit_, *x*^‡^<*w*_s_. (Note that we have assumed that *x*_DNA_⩾*w*_s_, which is always true in the present study. For DNA too short to span the width of the slit, *x*_DNA_<*w*_s_, and TST predicts *x*^‡^≤*x*_DNA_.)

We consider a DNA strand trapped in a nanogroove. Thermal fluctuations will now and then move one of its ends into the nanoslit (Fig. 2c,d). Let *x* denote the position of this end inside the nanoslit, measured in the direction parallel to the flow (Fig. 2d). This *x* is our reaction coordinate. Thus, *x*=0 denotes the equilibrium state of a DNA strand trapped in a nanogroove. Similarly, *x*^‡^ denotes the transition state for crossing into the next groove downstream. *x*=0 and *x*^‡^ correspond to local minima and maxima, respectively, of the free-energy landscape experienced by a DNA strand moving through the chip (Fig. 3a).

The drag force *f* pulling at the DNA in the slit is proportional to the length ℓ of this DNA, *f*=*γυ*_s_ℓ, where *υ*_s_ is the mean flow speed and *γ* is the effective drag coefficient of the DNA inside the slit (Fig. 2d). Since the flow in the chip is laminar, *υ*_s_ is proportional to the pressure drop over the microchip, Δ*P*, which we control experimentally. Assuming that ℓ is approximately proportional to the DNA's extension parallel to the flow, *x*, we find that the drag force on the part of the DNA strand inside the nanoslit is proportional to *x*Δ*P*. Thus, the decrease in free energy associated with the hydrodynamic drag force on the DNA is proportional to *x*^2^Δ*P*.

Note that the part of the DNA that rests in the nanogroove also experiences a drag force. It is proportional to the flow speed inside the nanogroove, *υ*_g_≈*υ*_s_/(1+*d*_g_/*d*_s_)=*υ*_s_/3. This force, however, does not contribute to the free-energy difference along the reaction coordinate, since the part of the DNA inside the nanogrove does not move downstream with the drag force it experiences in the groove.

The decrease in entropy caused by the introduction of the DNA into a nanoslit, where it is more confined than in a nanogroove, gives rise to an entropic recoil force that tends to pull the DNA back out of the slit. This decrease in entropy is proportional to *x* (ref. 24).

Introduction of an end of the DNA strand into a nanoslit thus changes its free energy by the amount 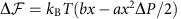 compared with its equilibrium state, where the whole strand resides in the nanogroove, *x*=0 (Fig. 3b)^19^,^20^,^24^,^25^. Here *a* and *b* are constants of proportionality that depend, respectively, on the mean drag coefficient on the DNA inside the nanoslit and the increase in entropy per unit length of DNA introduced into the nanoslit.

**Figure 4:**
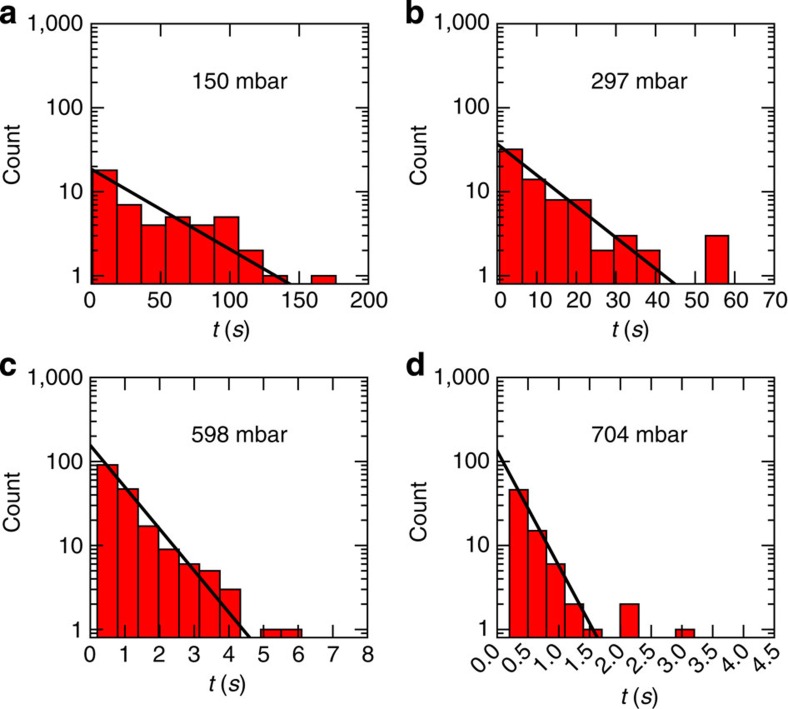
Example distributions of waiting times in a groove. Measured waiting times in nanogrooves separated by nanoslits of width *w*_s_=0.4 μm (chip 1 below) for different values of the pressure difference imposed over the chip, spanning most of the parameter range explored in experiments here. Histograms shown agree well with single exponential fits (solid lines—obtained from maximum likelihood estimation, see Methods). Apparent ‘outliers' arise from finite statistics in the tails and should be there, as their numbers agree with the expected numbers given by the areas under the tails of the theoretical distributions. Numbers of measured transition events are (**a**) 47, (**b**) 72, (**c**) 180 and (**d**) 73.

TST then predicts that the waiting times of a DNA strand in a groove are exponentially distributed (Fig. 4 and Supplementary Fig. 1) with a mean value that is given by *τ*=*τ*_0_ exp[*bx*^‡^−*a*(*x*^‡^)^2^Δ*P*/2]. Two regimes exist, separated by a critical pressure difference Δ*P*_crit_ (Fig. 3c): (i) a high-force regime, characterized by Δ*P*>Δ*P*_crit_, with the transition state inside the nanoslit at *x*^‡^=*b*/(*a*Δ*P*), and the dynamics of barrier crossing independent of *w*_s_; and (ii) a low-force regime, characterized by Δ*P<*Δ*P*_crit_=*b*/(*aw*_s_), with the transition state given by *x*^‡^=*w*_s_, where *w*_s_ is the width of the nanoslit.

**Figure 5:**
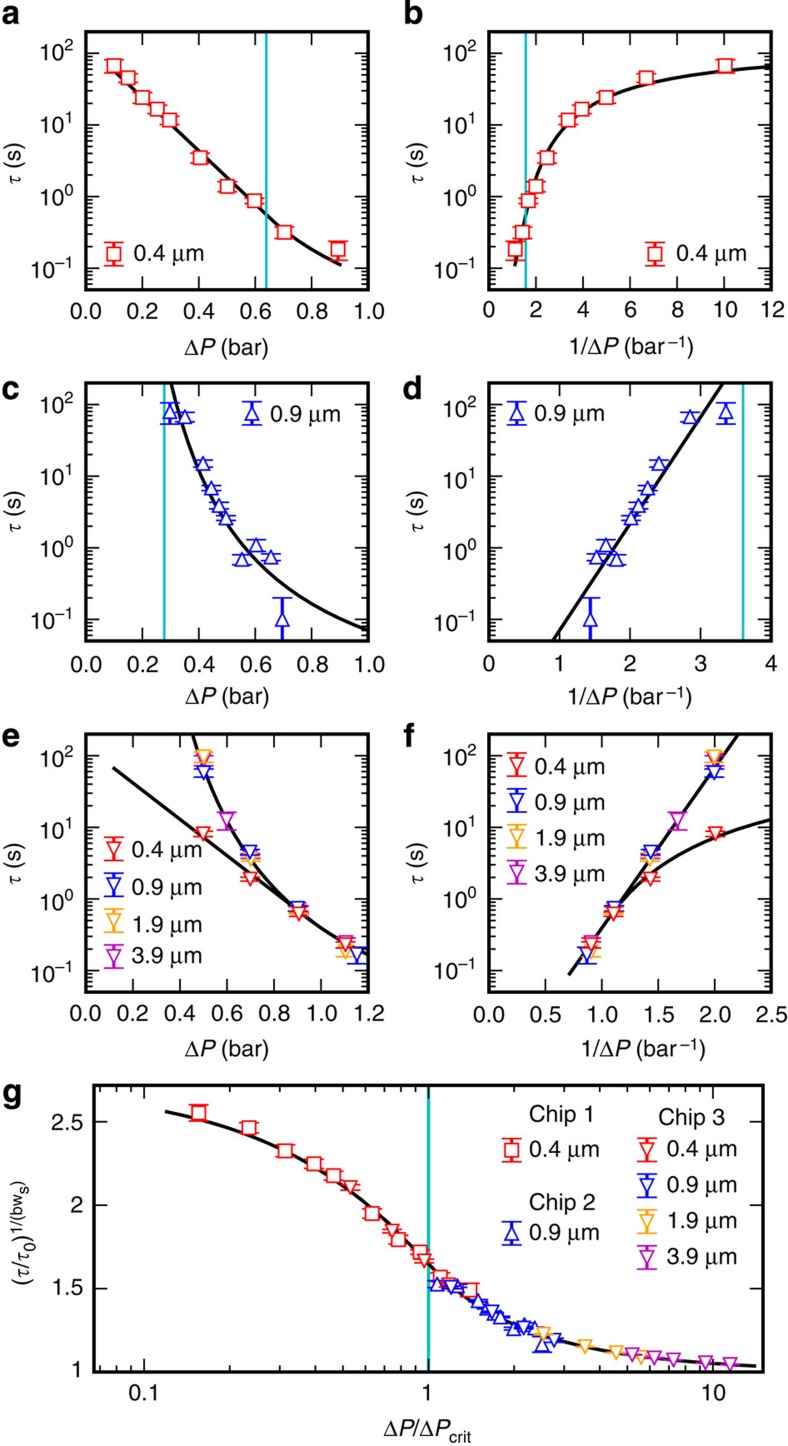
Average lifetimes of quasi-stationary states in various force fields. Experimentally measured average residence times *τ* of DNA strands in a nanogroove as function of the pressure drop Δ*P* over the microfluidic chip. Different symbols correspond to different chips, different colors correspond to different nanoslit widths, see legends; chip 3 has different nanoslit widths at different places and hence does several distinct experiments all on the same chip. Data were collected for three different chips: (**a**,**b**) chip 1 (642 transition events), (**c**,**d**) chip 2 (1,604 events) and (**e**,**f**) chip 3 (2,873 events). (**a**,**c**,**e**) *τ* as function of Δ*P*—data follow a straight line for Δ*P*<Δ*P*_crit_ and a hyperbolic curve for Δ*P*>Δ*P*_crit_; (**b**,**d**,**f**) *τ* as function of 1/Δ*P*—data follow a straight line for 1/Δ*P*<1/Δ*P*_crit_ and a hyperbolic curve for 1/Δ*P*>1/Δ*P*_crit_. Symbols: experimental data, mean±s.e.m. (examples of full distributions are shown in Fig. 4 and Supplementary Fig. 1); black lines: single fit of the theory (equation (2)) to all data in **a**–**f** (see Methods section); blue vertical lines: Δ*P*_crit_ for **a**,**c**,**e**, 1/Δ*P*_crit_ for **b**,**d**,**f**. (**g**) All data from the three microfluidic chips fall on a single curve given by equation (3), independent of chip geometry when expressed in dimensionless variables Δ*P*/Δ*P*_crit_ and 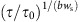.

The mean trapping time is thus given by




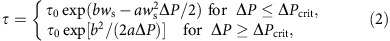




where the prefactor *τ*_0_ is related to the effective timescale of the motion along the reaction coordinate^1^,^2^,^3^,^4^. Equation (2) shows that for Δ*P*<Δ*P*_crit_, the trapping time is described by the Bell–Evans model and log(*τ*/*τ*_0_) is a first-degree polynomial in Δ*P* (Fig. 5a,c,e). For Δ*P*>Δ*P*_crit_, equation (2) shows that log(*τ*/*τ*_0_) is proportional to 1/Δ*P* (Fig. 5b,d,f), as also observed in refs 19, 20. At Δ*P*=Δ*P*_crit_ we have a continuous transition between the two distinct regimes (Fig. 5a,b). The values found for the parameters of equation (2) (Table 1) are connected with microscopic physical quantities. The value for *τ*_0_ suggest that the timescale of relaxation of the DNA inside the slit is of the order of milliseconds, while *a* and *b* are determined by the DNA's effective drag coefficient and persistence length, the degree of stretching and the effective confinement energy of the DNA inside the slit. The latter four quantities cannot be found from our values for *a* and *b* alone. But by using that the drag coefficient and persistence length for DNA under similar conditions were found to be *γ*≈1–2 fN s μm^−2^ and ℓ_p_≈40 nm, respectively, we can give rough estimates of the degree of stretching and effective confinement energy (see the section ‘Microscopic interpretation of parameter values' in Methods). We find that the degree of stretching of the DNA inside the slit is 30–50%, and the effective confinement energy of the DNA inside the slit is 0.4–0.6*k*_B_*T* per persistence length (ℓ_p_≈40 nm) of DNA introduced into the slit.

Finally, by renormalizing *τ* and Δ*P* as 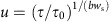 and *ξ*=Δ*P*/Δ*P*_crit_, we find that all data fall on the same curve (Fig. 5g) given by




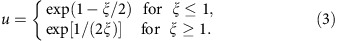




### Why TST works here

We made several simplifying assumptions to derive equations (2) and (3). These assumptions hold for our experiments for the following reasons.

(i) We assume that the DNA strand is in a state of thermal quasi-equilibrium when trapped in a nanogroove with its ends occasionally, randomly entering the nanoslit—more specifically, each point of the free-energy landscape inside the trap should be visited with a probability given by its Boltzmann factor. The validity of this assumption depends on the timescale of relaxation of the DNA strand in a trap, *τ*_relax_, being much shorter than the average time to escape from the trap.

For *λ*-DNA in a nanogroove with cross-section ≈100 × 150 nm^2^ (similar to here), *τ*_relax_∼1 s (ref. 26). Inside the slit, the relaxation time is much smaller than this due to additional confinement^26^, the tension on the strand due to drag and the short length of DNA that is inside the slit (the relaxation timescales as ∼1/*L*)^26^. Confinement reduces the relaxation time by a factor ∼5 (ref. 26). Less than one-tenth of the DNA is in the slit before the transition state is traversed, as the longest distance separating the initial and transition states is 0.8 μm, further reducing *τ*_relax_ in the slit by a factor ∼10.

These two effects alone then reduce the relaxation time to ∼10 ms, while drag reduces it further, in agreement with the fitted value of *τ*_0_ being of the order of milliseconds. This is fast enough compared with typical waiting times and experimental resolution that we may consider the DNA to be in quasi-equilibrium in the energy landscape before crossing the barrier; the exponential distribution of recorded waiting times to escape confirms this (Fig. 4 and Supplementary Fig. 1), while any ‘inertial' effects making a second transition more probable immediately after a transition has occurred, say, due to incomplete relaxation in the trap, would result in an excess of counts for low waiting times.

(ii) We do not need the no-recrossing assumption, since Kramers' problem with large friction covers our case. It is approximately valid, however, if the free-energy landscape is steep and drops far, starting just past the transition state, and this condition is satisfied in our experiment.

Consider the case of lowest force (*w*_s_=0.4 μm and Δ*P*=100 mbar), where the free-energy landscape is the least steep. The height of the free-energy barrier here is 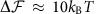, and the slope of the energy landscape to the right of the transition state is ≈−5*k*_B_*T* per μm. Since recrossing happens with a probability that is proportional to the Boltzmann factor, *x* needs not be much larger than *x*^‡^ for recrossings to become highly unlikely, and translocation is hence effectively irreversible in our experiments.

Our data confirm this understanding. Recrossings over the transition state leads to ‘dynamical corrections' of *τ*_0_: it is expected to scale with the width of the free-energy barrier^4^. For Δ*P*≫Δ*P*_crit_, the barrier is essentially a parabolic potential, and one finds *τ*_0_∼Δ*P*^−1/2^, while for Δ*P*<<Δ*P*_crit_, the landscape is essentially rectilinear around the barrier, and one finds *τ*_0_∼Δ*P*^−1^ (ref. 4). For our experiments, this dependence on Δ*P* is so weak compared with the exponential factor in equation (2) that Fig. 5 shows agreement between data and theory without these dynamical corrections.

Further assumptions used here are the following: the shape of the free-energy landscape of the DNA in the nanoslit was derived assuming (iii) that nonlinear effects of hydrodynamic self-screening of the DNA in the slit is negligible; (iv) that the degree of stretching of the DNA in the slit does not depend on the amount of DNA in the slit; (v) that the increase in confinement energy caused by introduction of an end of the DNA into the slit scales linearly with the amount of DNA contour introduced; and (vi) that escape over the barrier through formation of a hernia, that is, a hairpin-like protrusion, of DNA in the slit happens so rarely that it can be ignored.

The effects of screening (iii) and uneven stretching (iv) tend to cancel each other, while both are diminished by the high degree of stretching of the DNA inside the nanoslit.

Assumption (v) that the free-energy cost per unit length of escaping contour is constant can be justified by noting that the free energy of confinement 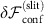 and 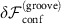 both scale linearly with contour present in the slit and groove, respectively. Thus, the cost in free energy per unit length of contour, 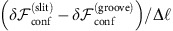, of driving contour length Δℓ from the groove to the slit is constant. This linear scaling is fundamental and will hold regardless of the specific confinement regime as long as the size of the slit- or groove-confined polymer is much larger than the size of a ‘statistical blob' in the slit or groove (which is true for the *λ*-DNA used here). To see this, simply note that for a confined polymer of contour length *L*, with *k*_B_*T* stored per blob, 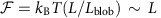. For a semiflexible chain, this linear scaling of confinement free energy with contour has been explicitly demonstrated in ref. 27 (for a slit) and ref. 28 (for a nanochannel, which approximates a nanogroove geometry well in as much as both force DNA into linearly extended configurations).

Finally, we assumed (vi) that escape over the entropic barrier through formation of a hernia inside the slit is an event sufficiently rare to be ignored. This escape event would lead to a non-exponential distribution of waiting times since the timescale of escape via this mechanism differs from that of end-induced escape. The reasons we do not observe escape via hernias here are threefold. First, as a hernia may form anywhere along a DNA strand, the rate of escape via hernias scales linearly with the length of the DNA^19^,^21^,^29^,^30^. Since *λ*-DNA is relatively short, this suppresses escape via herniation. Second, the free energy of a herniation inside the slit is more than twice that of an end (for an ideal flexible chain, it is exactly twice as high)^29^,^30^. So the probability of finding a hernia (as opposed to its multiplicity) is the square of the probability of finding a given end extending equally far into the slit. The latter being small, the former is very small. Third, since the DNA in the groove is stretched and the timescale of herniation is much faster than DNA relaxation in the groove (compare *τ*_0_∼1 ms with *τ*_relax_∼1 s), introduction of a hernia into the nanoslit must stretch the DNA in the groove close to the hernia, thus increasing the free-energy barrier against escape via herniation even more. In contrast, introduction of an end into the nanoslit does not decrease the entropy per unit length of the DNA remaining in the groove.

## Discussion

The use of an external pressure gradient to control and understand translocation of molecules by nanofluidic flows is poorly represented in the literature. So are simple models of such processes and their experimental verification.

Here we have shown that TST describes translocation of DNA driven by a hydrodynamic flow through a nano-confined environment that forms a series of entropic traps. We observed two distinct regimes: (i) a high-force regime in which the free-energy barrier is parabolic around the transition state. The transition state consequently moves along the reaction coordinate when the external force is altered; and (ii) a low-force regime in which the transition state is sharp and thus does not move when the force is altered. Observation of this low-force regime was made possible by reducing the barrier width considerably compared with earlier experiments^19^,^20^,^22^. A simple order-of-magnitude calculation shows that one would have to wait on the order of 100 years to see a single translocation in the geometries used in refs 19, 20, 22 (see the section ‘Size matters' in Methods).

The applicability of TST to DNA translocation over entropic barriers relies on two conditions on the energy landscape describing the barriers: (i) the barrier separating two traps must be sufficiently high for quasi-equilibrium to exist before translocation; and (ii) the barrier must be steep enough beyond the transition state for recrossing not to occur, effectively.

If (i) is not satisfied, for example, for very high force, the motion becomes (partially) ballistic^21^, leading to a non-exponential distribution of waiting times with an excess of short waits. If (ii) is not satisfied, for example, for very low force, Brownian motion dominates over drift, so the escape process is no longer irreversible^23^ and hence ill defined as an ‘escape.'

Microscopic ‘bottom-up' models for barrier crossing of ideal Rouse polymers (polymers without bending rigidity and excluded volume effects)^29^,^30^,^31^,^32^,^33^ yield an expression (equation (25) of ref. 30) for the rate of barrier crossing that is similar to our simple TST result (equation (1)). In these models the polymer crosses the barrier by stretching out in a ‘kink' configuration, if it is long enough, since this lowers its free energy. An experimental demonstration of such stretching is provided in ref. 34, which shows DNA stretching where it crosses a potential barrier created by a conservative thermophoretic force field.

Note, however, that the physical mechanism responsible for the stretching of DNA in our nanoslit is entirely different. In refs 29, 30, 31, 32, 33, and in essence also in ref. 34, each monomer experiences the same potential energy barrier. Stretching lowers the energy barrier for the whole polymer by placing fewer monomers on top of the barrier, while stretching also costs a decrease in entropy, since some degrees of freedom are suppressed. The polymer stretches across the barrier when this decreases its potential energy by more than the ensuing cost in entropic free energy. However, this stretching is just a side effect of having the potential energy barrier; it is an adaption of the polymer to it. The barrier remains a potential energy barrier, while it affects the configurational entropy of the polymer.

In our experiments, on the other hand, DNA entering the nanoslit is less free to move thermally than it is in the nanogroove. The ensuing cost in entropic free energy constitutes the whole free-energy barrier. Thus, this barrier is entropic in nature. (Note that Figs 4 and 5 in ref. 30 resemble ours, but are artefacts resulting from displaying the DNA in an extra, non-existing dimension.) The final results are similar, because it is only the total free energy (containing both the energetic and entropic contributions) that matters. It should be interesting to extend the models of refs 29, 30, 31, 32, 33 to a non-smooth energy landscape to see if these microscopic models can predict the crossover between the high- and low-force regimes observed here.

The methods developed here might be useful also in the study of translocation of other biomolecules with more complex topologies (RNA, proteins, circular DNA or branched polymers) and in biological phenomena such as chromatin translocation in the cell nucleus and nuclear export. In particular, the quadratic shape of the energy landscape seen here is predicted also for more complex polymers, by scaling arguments^25^,^35^. It would be interesting to investigate experimentally whether in consequence the statistics of barrier crossing for such more complex polymers also is described by the simple formulas derived here.

The study presented here demonstrates the wide applicability of TST and the Bell–Evans model, in particular to out-of-equilibrium transport in confined environments. It is, to the best of our knowledge, the first time that the Bell–Evans model for barrier crossing under external load has been demonstrated on the micron scale and for polymers crossing entropic barriers. The fact that the process can be monitored with video microscopy should appeal to anyone who teaches or has been taught TST.

In general, our study may serve as a reminder that TST applies where thermal activation is possible, irrespective of length scales. Its rates are dominated by the factor given in equation (1) above, so the Boltzmann energy sets the scale, while length scales are irrelevant in a first approximation. This physics insight can be used deliberately in engineering, in microfluidic handling of polymers, particles or cells. Or, if ignored, it might cause problems.

This fact that TST applies on any length scale where thermal activation is possible, is a small demonstration of the universality of many physical theories. TST is more universal than that, however: The Brownian motion at its core needs not be thermal in origin^36^, so TST can describe other random processes as well.

## Methods

### Device fabrication and experimental set-up

The nanofluidic devices were fabricated from fused silica wafers (JINSOL)^37^. Electron beam lithography in zep520A resist was used to define the nanogrooves, and photolithography in AZ5214E resist was used to define the nanoslit and the inlet channels. The structures were transferred to the silica through CF_4_:CHF_3_ reactive ion etching, and the channels were closed by fusion bonding of a 157-μm-thick fused silica coverslip to the wafer surface. Experiments were performed using *λ*-phage DNA (48.5 kb, New England Biolabs) stained with the fluorescent dye YOYO-1 (Molecular Probes) at a ratio of 1 dye molecule per 5 base pairs. A buffer solution of 0.5 × TBE (0.445 M Tris-base, 0.445 M boric acid and 10 mM EDTA) with 3% beta-mercaptoethanol was used. The buffer was driven through the nanogroove channel by applying air pressure, controlled to a precision of 1 mbar, to the inlets of the device. The DNA molecules were observed using an epi-fluorescence microscope (Nikon Eclipse TE2000-U) with × 60 and × 100 oil immersion 1.4 NA objectives. Movies of the DNA were recorded at up to 10 fps with an electron multiplying charge-coupled device camera (Cascade II, Photometrics). Identification of DNA molecules in the sidewinder state was performed using two distinct criteria^21^: (i) the DNA rested at least two frames in a groove between two transitions; and (ii) during a transition, both the DNA contour that connects two grooves and the DNA inside each groove were stretched, along the flow direction and the nanogroove, respectively. Waiting times were defined as the durations between the time at which the DNA found its equilibrium configuration inside a nanogroove after a transition and the time at which the leading end of the DNA had crossed over into the next nanogroove. In total, 5,119 sidewinder transitions were observed (chip 1: 642; chip 2: 1,604; and chip 3: 2,873).

### Length and persistence length of YOYO-1-stained DNA

Intercalating YOYO dye affects both the length *L* and the persistence length ℓ_p_ of DNA. The dye increases *L* by approximately a factor 1.3 for a concentration of 1 YOYO-1 molecule per 5 base pairs^38^. For *λ*-DNA this leads to *L*≈21 μm. There is controversy in the literature as to its effect on ℓ_p_, however, with some studies reporting an increase^39^,^40^ in ℓ_p_ and others^38^,^41^,^42^ reporting a decrease in ℓ_p_. We here use the most recent results of ref. 38, which, for a dye concentration of 1 YOYO-1 molecule per 5 base pairs, gives ℓ_p_≈40 nm.

Note that using the results of refs 39, 40, which give ℓ_p_=65 nm, does not change the conclusions presented in the sections ‘Microscopic interpretation of parameter values' and ‘Size matters' below. The following argument shows this.

For ℓ_p_≈65 nm, we find from our estimated value of *b* an effective confinement energy per persistence length inside the nanoslit of 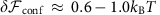, which should be compared with 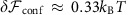 from equations (6) and (7). The change of entropy due to the introduction of one persistence length of DNA into the nanoslit in the microarray of refs 19, 20 is found to be 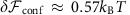 and 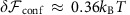 for ref. 22. For ℓ_p_≈65 nm, the confinement energy in the geometries of refs 19, 20, 22 would thus be roughly twice as high as for ℓ_p_=40 nm, and the argument used below would then give that the expected waiting time for a single DNA strand to cross the barrier at critical field strength is ∼10^10^ years or more.

### Calculating the mean of exponentially distributed data

As described in ref. 21, the DNA may at random switch from the ‘sidewinder state', in which it is trapped for a time *t*_*i*_ in thermal equilibrium in a nanogroove, before it escapes to the next nanogroove, to a ‘tumbleweed state', in which the DNA strand moves through the array without getting trapped in the grooves. Thus, there are no waiting times in the tumbleweed state; motion is continuous. The interaction with a groove may, however, slow the speed of the DNA in the tumbleweed state. This may falsely be detected as a waiting time by our movie-analysis software. As a filter against such false positives, we discard all waiting times that are shorter than twice the time Δ*t* between frames in the movie. This means, however, that we also reject some true positives: DNA strands that are in the sidewinder state, but escape sooner than 2Δ*t*. Thus, the average 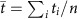 of the measured dwell times is a biased estimate of 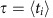. Instead we use the unbiased (maximum likelihood) estimator 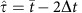.

### Parameter estimation

Equation (2) is our theory for the observed waiting times. We fit the parameters *θ*=(*a*_1_, *a*_2_, *a*_3_, *b*, *τ*_0_) of this theory to data 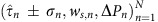 using weighted least squares with weights 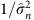. Here 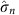 is the empirically estimated s.e.m. of 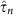. We fit simultaneously to the data from all three microchips. In this fit, the parameters (*a*_1_, *a*_2_, *a*_3_) are allowed individual values for each chip, since they depend on the hydraulic resistance, which differs between chips. The parameters *b* and *τ*_0_ depend only on the DNA, solvent and temperature. They should not differ between chips, so we fit values shared by all chips. Fitting to data presented in Fig. 5, we obtain the estimates given in Table 1.

The variances of errors on fitted parameter values were estimated as




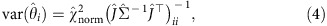




where 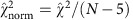 is 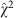 per degree of freedom, 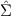 is the covariance matrix of 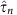 with entries given by 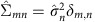 and 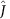 is the Jacobian of the vector 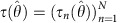, with entries given by 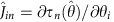.

### Microscopic interpretation of parameter values

The parameter values returned by the fit described above can be interpreted at the microscopic level. Thus, the value obtained for *τ*_0_ means that the effective timescale of DNA motion in the free-energy potential inside the slit is of the order of milliseconds.

The values found for *a* is connected with the degree of stretching of the DNA inside the nanoslit. From our definition of *a* and *x*, we have *γυ*_s_ℓ^2^/(2*k*_B_*T*)≈*a*Δ*Px*^2^/2. The mean degree of stretching of the DNA inside the slit is then given by




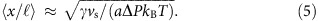




Here *υ*_s_≈Δ*P*/(*d*_s_*w*_ch_*R*_hyd_), where *w*_ch_=50 μm is the chip width perpendicular to the flow, and 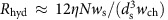 is the hydraulic resistance over the chip^43^, where *N* is the number of slits in the chip and *η* is the dynamic viscosity of water. Since equation (5) depends on the value of *γ*, our estimate for *a* does not directly give us the degree of stretching of the DNA inside the nanoslit. However, the drag coefficient for a flow parallel to the DNA backbone has been found previously for similar conditions to be *γ*_||_≈1 fN s μm^−1^ (J.N. Pedersen, personal communication). For DNA segments aligned perpendicular to the flow, 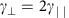, so we expect the effective drag coefficient on DNA inside the slit to lie between these two values.

We use this range of values for *γ* in the following and that *η*=10^−3^ Pa·s, *k*_B_*T*=4.1 fN μm and *d*_s_=0.05 μm. For chip 1 we have *N*=900, *w*_s_=0.4 and *a*=0.11, yielding 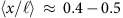. For chip 2 we have *N*=450, *w*_s_=0.9 and *a*=0.11, yielding 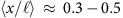. For chip 3 we have *N*=375, average slit width 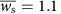 and *a*=0.07, yielding 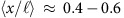.

Finally, we may use the above estimate of the degree of stretching inside the nanoslit and the value of *b* to give a rough estimate of the effective confinement energy of the DNA inside the slit compared with the groove. Since *b* gives the effective confinement energy in the slit per μm along the reaction coordinate, we may find the confinement energy per persistence length ℓ_p_ as 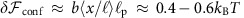 by using ℓ≈40 nm and the values for 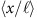 found above.

We may compare this result with the expected difference in confinement energies between the slit and groove, 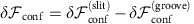. The DNA in the groove and in the slit is in a crossover regime between the De Gennes and Odjik regimes. Here interpolation formulas for the confinement energy were recently determined from a combination of high precision simulations and experiments. The free energy of confinement per persistence length of DNA in a channel (which approximates the nanogroove geometry) is^28^




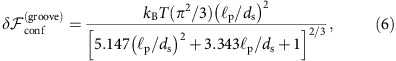




while the confinement energy in the nanoslit is^27^,^44^




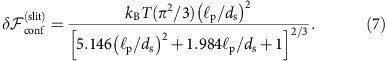




The expected confinement energy per ℓ_p_ is thus 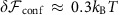, which is somewhat smaller than the energy estimated from *b*. This difference may be explained by the flow stretching the DNA inside the slit, such that *b* reflects not only the confinement energy due the walls of the slit but also contains a term from the stretching of the DNA due to the flow. Table 1 collects our estimates for microscopic quantities.

### Size matters

In the present experiment, the barrier width is significantly smaller than in earlier similar experiments^19^,^20^,^22^. This makes all the difference for our ability to observe the low-force regime (Δ*P*<Δ*P*_crit_).

In refs 19, 20, the potential wells of the microarray with the smallest dimensions were separated by *w*_s_≈2 μm of nanoslit, while (ref. 22) studied DNA translocation through nanopores of 6 μm or more. This made observation of the low-force regime impossible according to the following order-of-magnitude estimate of the time—100 years or more—that one would have to wait for a single DNA molecule to traverse the nanoslit/nanopore in these geometries. Our argument hinges on the fact that the mean trapping time of a DNA strand at critical pressure difference Δ*P*_crit_ depends exponentially on *bw*_s_, that is, *τ*_crit_=*τ*_0_ exp(*bw*_s_/2). Thus, a linear increase in *b* or *w*_s_ leads to an exponential increase in the waiting time.

The entropic traps (microgrooves) of refs 19, 20 had dimensions *d*_g_≈1 μm by *w*_g_≈2 μm. Since the radius of gyration, *R*_*g*_≈0.5 μm, of YOYO-stained *λ*-DNA is smaller than both *d*_g_ and *w*_g_, the DNA was essentially in bulk conformation there. The increase in free energy due to confinement caused by the introduction of a unit length ℓ of DNA from bulk into the nanoslit was thus simply equal to its confinement free energy in the slit, given by equation (7).

For a height of *d*_s_≈0.09 μm of the nanoslits of refs 19, 20, this gives 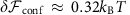, which is comparable to the confinement energy in our set-up of 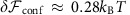 found from equations (6) and (7). So while the DNA strand is more confined in the nanoslit in our set-up than in the one of refs 19, 20 (*d*_s_=50 nm in our case versus *d*_s_≈90 nm in refs 19, 20), the preconfinement in the nanogroove lowers the relative confinement energy here such that the entropic recoil force is similar in magnitude to, or lower than, the one in refs 19, 20.

In the present experiment, we have *τ*_crit_≈0.5 s from *τ*_0_=0.002 s and *b*=27 μm^−1^, found by fitting equation (2) to data (see ‘Parameter estimation' above). We assume that *τ*_0_ does not change much for different geometries, and since 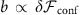, we may assume that *b* was roughly the same in the set-up of refs 19, 20 as here. Thus, since their narrowest nanoslit measured 2 μm, the expected mean waiting time at critical field strength would in their geometry be *τ*_crit_∼100 years. Similarly, for the least constricted geometry of ref. 22, we have 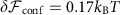 (approximating their nanopore as a nanochannel), that is, around half of the confinement energy of the present study. However, since their pore measured 10 μm, we find here *τ*_crit_∼10^20^ years.

## Additional information

**How to cite this article:** Vestergaard, C. L. *et al*. Transition state theory demonstrated at the micron scale with out-of-equilibrium transport in a confined environment. *Nat. Commun.* 7:10227 doi: 10.1038/ncomms10227 (2016).

## Supplementary Material

Supplementary Information Supplementary Figure 1

## Figures and Tables

**Figure 1:**
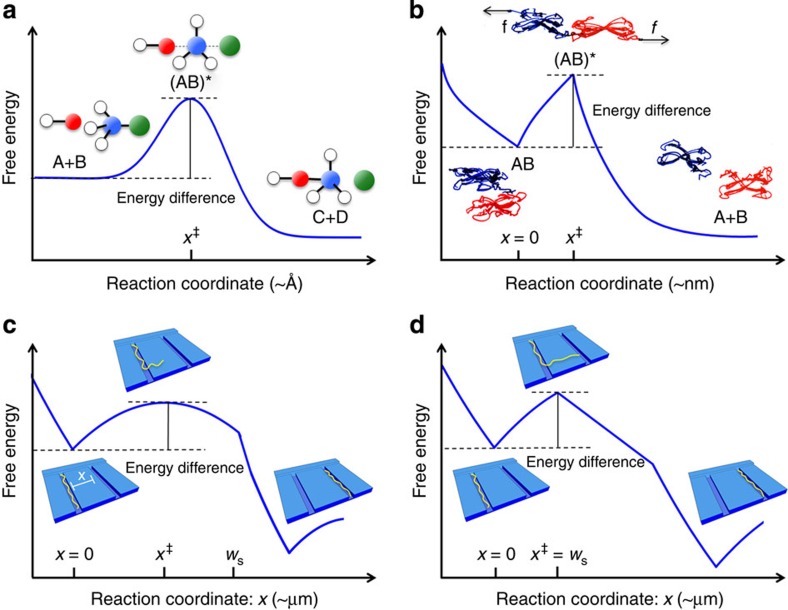
The scenario of TST. A free-energy landscape is traversed by a reaction pathway that is parameterized by a reaction coordinate;^1^,^2^ typical length scales of reaction pathways are given in parentheses. Insets portray physical situations corresponding to (quasi) equilibrium and transition states (at *x*^†^). (**a**,**c**) Transition states that will change location on the reaction pathway with the strength of an applied force, exemplified by (**a**) a chemical reaction (with transition state (AB)*), and in the present study (**c**) a polymer crossing an entropic barrier in the form of nanoslit separating two nanogrooves, where the transition state lies inside the nanoslit. (**b**,**d**) Sharp transition states that are independent of an applied force, similar to the situation in chemical bond rupture; here exemplified by (**b**) the separation of two binding proteins under an external force (with transition state (AB)*), and in the present study (**d**) a polymer crossing through a nanoslit where the transition state is located at the end of the nanoslit. In **c**,**d** the reaction coordinate *x* parameterizes the continuous shifting of DNA in a transition from the upstream to the downstream nanogroove. Specifically, it measures the extension of the leading end of the DNA into the nanoslit, until this end of the DNA enters the next nanogroove, which happens at *x*=*w*_s_. For 0<*x*<*w*_s_, *x* is approximately proportional to the contour length ℓ of the DNA that has left the upstream nanogroove. After the leading end of the DNA has entered the downstream nanogroove, we let *x* denote a fixed fraction of the contour length ℓ of the DNA that has left the upstream nanogroove, the same fraction as *x* denoted for 0<*x*<*w*_s_. Note that we need not know the value of this fraction, and its existence can be an approximation. The qualitative picture described here still captures the essence of Fig. 2's experimental observations of transitions. The trailing end of the DNA leaves the upstream nanogroove when ℓ equals the full contour length of the DNA molecule, *L*_DNA_. We denote the value of *x* at that point by *x*=*x*_DNA_. After this point, we let *x* denote *x*_DNA_ plus the distance that the DNA molecule's trailing end has moved into the nanoslit. After the DNA has completely entered the next nanogroove, the landscape repeats itself as from *x*=0 (see also Fig. 3).

**Table 1:** Estimated parameter values (top) and estimated values of related microscopic quantities (bottom).

***τ***_**0**_ **(ms)**	***a***_**1**_ **(μm** ^−2^ ** mbar** ^−1^ **)**	***a***_**2**_ **(μm** ^−2^ ** mbar** ^−1^ **)**	***a***_**3**_ **(μm**^−2^ **mbar**^−1^**)**	***b*** **(μm** ^−1^ **)**
2.2 (68% CI (0.6, 7.6))	0.11±0.03	0.11±0.01	0.07±0.01	27±4
	〈*x*/ℓ〉	〈*x*/ℓ〉	〈*x*/ℓ〉	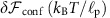
	0.4–0.5	0.3–0.5	0.4–0.6	0.4–0.6

CI, confidence interval.

Microscopic quantities were estimated using *γ*=1–2 fN s μm^−2^ and ℓ_p_=40 nm as described in the section ‘Microscopic interpretation of parameter values' in Methods.
