# Supplementary material for: Transition state theory demonstrated at the micron scale with out-of-equilibrium transport in a confined environment
Source: Nat Commun. 2016 Jan 6;7:10227. doi: 10.1038/ncomms10227 (PMC5154429; doi:10.1038/ncomms10227)
Supplement: Supplementary Information — Supplementary Figure 1 [file ncomms10227-s1.pdf]

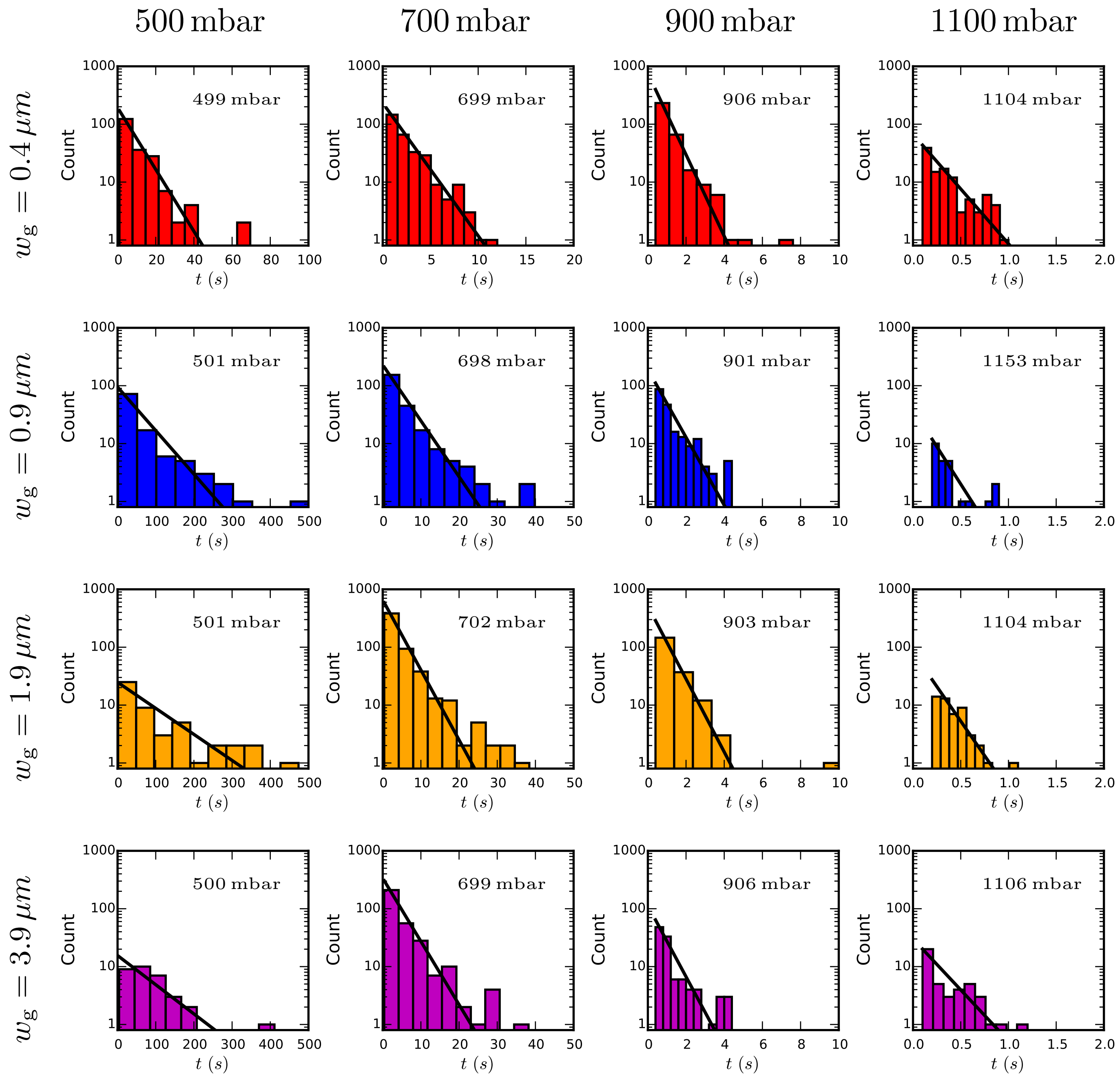

Supplementary Figure 1: **Examples of measured waiting time distributions.** Distributions of waiting times from which average waiting times of Fig. 5e,f (Chip 3) were calculated. Full lines show MLE fits of a single exponential with a cutoff at $t = 2\Delta t$. Each row corresponds to a different nanoslit width as indicated on the left. Measured pressure drop over the chip is indicated in each panel. Colors correspond to those of Fig. 5. For $\Delta P = 900\,\mathrm{mbar}$ and $\Delta P = 1100\,\mathrm{mbar}$, the scale of the distributions for $p_{\rm g} = 0.4\,\mu m$ is similar to those for $p_{\rm g} \geq 0.9\,\mu m$, while for $\Delta P = 500\,\mathrm{mbar}$ and $\Delta P = 700\,\mathrm{mbar}$, it differs; this is indicative of a crossover between high- and low-force regimes (Fig. 5e,f).
